# Correction: Inhibition of CERS1 in skeletal muscle exacerbates age-related muscle dysfunction

**DOI:** 10.7554/eLife.99846

**Published:** 2024-05-20

**Authors:** Martin Wohlwend, Pirkka-Pekka Laurila, Ludger JE Goeminne, Tanes Imamura de lima, Ioanna Daskalaki, Xiaoxu Li, Giacomo von Alvensleben, Barbara Crisol, Renata Mangione, Hector Gallart-Ayala, Amelia Lalou, Olivier Burri, Stephen Butler, Jonathan C Morris, Nigel Turner, Julijana Ivanisevic, Johan Auwerx

**Keywords:** *C. elegans*, Human, Mouse

Wohlwend M, Laurila P-P, Goeminne LJE, Lima T, Daskalaki I, Li X, von Alvensleben G, Crisol B, Mangione R, Gallart-Ayala H, Lalou A, Burri O, Butler S, Morris J, Turner N, Ivanisevic J, Auwerx J. 2024. Inhibition of CERS1 in skeletal muscle exacerbates age-related muscle dysfunction. *eLife* **12**:RP90522. doi: 10.7554/eLife.90522.

Published 20 March 2024

One of the analyses in the final paper comprises *C. elegans* skeletal muscle morphology experiments upon Cers1 inhibition (Figure 4E). This experiment was conducted by Amélia Lalou at École Polytechnique Fédérale de Lausanne (EPFL). During the review process, Amélia Lalou was accidentally removed from the author list. We are therefore formally correcting the eLife paper to include Amélia Lalou in the author list. She has reviewed the manuscript, supports the conclusion and agrees to be responsible for all the parts of the paper in its published form. All authors have agreed to the change.

The details of Amélia Lalou’s contributions are as follows:

Amélia Lalou was instrumental in conducting the confocal muscle fiber imaging of P053 treated myo3p *C. elegans* worms. Amélia Lalou has reviewed the manuscript and its conclusions.

**New author list:**

Martin Wohlwend, Pirkka-Pekka Laurila, Ludger JE Goeminne, Tanes Lima, Ioanna Daskalaki, Xiaoxu Li, Giacomo von Alvensleben, Barbara Crisol, Renata Mangione, Hector Gallart-Ayala, Amélia Lalou, Olivier Burri, Stephen Butler, Jonathan Morris, Nigel Turner, Julijana Ivanisevic, Johan Auwerx

**Original author list:**

Martin Wohlwend, Pirkka-Pekka Laurila, Ludger JE Goeminne, Tanes Lima, Ioanna Daskalaki, Xiaoxu Li, Giacomo von Alvensleben, Barbara Crisol, Renata Mangione, Hector Gallart-Ayala, Olivier Burri, Stephen Butler, Jonathan Morris, Nigel Turner, Julijana Ivanisevic, Johan Auwerx

**Details for the omitted author:**

Amélia Lalou

Laboratory of Integrative Systems Physiology, École Polytechnique Fédérale de Lausanne, Switzerland

**Contribution:** Data curation

**Competing Interests:** AL declares no competing interests.

The article has been corrected accordingly.

