# Supplementary material for: Inhibition of CERS1 in skeletal muscle exacerbates age-related muscle dysfunction
Source: eLife. 2024 Mar 20;12:RP90522. doi: 10.7554/eLife.90522 (PMC10954306; doi:10.7554/eLife.90522)
Supplement: Supplementary file 1. [file elife-90522-supp1.docx]

**Supplementary File 1.** Cellpose training quality control.

| Image | Prediction v. GT Intersect over Union | False positive | True positive | False negative | Precision |
| --- | --- | --- | --- | --- | --- |
| Image_LIS-123_region_0.tif | 0.810790951 | 4 | 148 | 16 | 0.973684 |
| Image_LIS-308_region_0.tif | 0.747845652 | 4 | 68 | 27 | 0.944444 |
| Image_LIS-140_region_0.tif | 0.906795138 | 4 | 129 | 3 | 0.969925 |
| Image_LIS-301_region_0.tif | 0.881344966 | 2 | 258 | 9 | 0.992308 |
