# Supplementary material for: Inhibition of CERS1 in skeletal muscle exacerbates age-related muscle dysfunction
Source: eLife. 2024 Mar 20;12:RP90522. doi: 10.7554/eLife.90522 (PMC10954306; doi:10.7554/eLife.90522)
Supplement: Supplementary file 2. [file elife-90522-supp2.docx]

**Supplementary File 2.** List of mouse and human qPCR primers.

| Gene symbol (mouse) | Forward | Reverse |
| --- | --- | --- |
| *Myod1* | AGCACTACAGTGGCGACTC | GTGGAGATGCGCTCCACT |
| *Pax7* | TCTCCAAGATTCTGTGCCGAT | CGGGGTTCTCTCTCTTATACTCC |
| *Myf5* | TGAGGGAACAGGTGGAGAAC | TGGAGAGAGGGAAGCTGTGT |
| *Myf6* | AGATCGTCGGAAAGCAGC | CCTGGAATGATCCGAAACAC |
| *Myog* | TTGCTCAGCTCCCTCAACCAGGA | TGCAGATTGTGGGCGTCTGTAGG |
| *Myh4* | ACAAGCTGCGGGTGAAGAGC | CAGGACAGTGACAAAGAACG |
| *Myh1* | CCAAGTGCAGGAAAGTGACC | AGGAAGAGACTGACGAGCTC |
| *Myh2* | CAGAGGCAAGTAGTGGTGGA | CAAATTCTCTCTGAACAGGGCA |
| *Myh4* | ACACAGAGTCAGGCGAGTTT | CAGTGCGTTCTTGGCCTT |
| *Myh7* | GTGGCTCCGAGAAAGGAAG | GAGCCTTGGATTCTCAAACG |
| *Myl1* | AGAGGTAGAAGCGTTGCTGG | GGCCAGTCTTCCCCAACATT |
| *Cers1* | CCACCACACACATCTTTCGG | GCCTGACCTCCAGTCATAGA |
| *Sptlc2* | GCACTCGTCAGGAAATTGGAAA | CTCCTAGAACCAGTGACGCA |
| *Degs1* | AATGGGTCTACACGGACCAG | GGACGAGAAGCATCATGGCTA |
| *Cers2* | GGCGCTAGAAGTGGGAAAC | TCGAATGACGAGAAAGAGCA |
| *mTorc1* | GGCATAACAGATCCTGACCCTGAT | CAGGGATGCCAAGACACAGTA |
| *Rps6kb1* | CGGGTACTTGGTAAAGGGGG | ATGTTCCGCTCTGCTTTCGT |
| *Eif4ebp1* | GGGGTCACTAGCCCTACCA | TCATTGCGTCCTACGGCTG |
| *Mstn* | TCACGCTACCACGGAAACAA | TGCCATCCGCTTGCATTAGA |
| *Smad3* | CACTCCCCAACCAGCTCAG | CAACTGCCTGAACATCTCCTCT |
| *Foxo1* | AAGGATAAGGGCGACAGCAA | ATTTTCCGCTCTTGCCTCCC |
| *Atrogin-1* | TCTCTCCTGGAAGGGCACTG | TGAGGGGAAAGTGAGACGGA |
| *Murf* | CACAGAGGGTAAAGAAGAACACC | TGGGGAGCCCTATGCTAGTC |
| *Ube2e1* | GTTGCTGCACTTCCGCTTC | GCGAACGGCCCCTCTCTTT |
| *MYOD* | TCTCCTTGGTGTAGGCTCAG | CCTGACCTTGAACGTGAATC |
| *MYOG* | TTGCTCAGCTCCCTCAACCAGGA | TGCAGATTGTGGGCGTCTGTAGG |
| *MYF5* | TGTGGCTCTCTCTCCGTATG | AATACAGACATGCAGGCTTCAC |
| *MYF6* | GTGGAGGAAGTGGTGGAGAA | ACTTTTCGGTCTGGGTTCCT |
| *MYH1* | TGTCTCCAAAGCCAAGGGAAA | CCCTCGAGAGCTGTGAAACT |
| *MYH2* | GTCCTGCTTTAAAAAGCTCCAAGA | TCAAAGGGCCTATTCTGGGC |
| *MYH7* | TTGGCCCCTTTCCTCATCTGT | ATCAGGCACGAAGACATCCTT |
| *MYH4* | GCTGAAGAGGCTGAGGAACA | CCCGACTCTTCACTCTCAGC |
| *MYL1* | AACCACCACTCCTCTTCCAA | AGGGTGGGTTAAAAAGAGAAGGA |
| *CERS1* | TCGTCTCCTCCTACGCCTTC | GCGGAACCAGAACCAGC |
| *SPTLC1* | AGTGGGTTCTGGTGGAGATG | TGGTAAGCAGGAGCCTCGTAA |
| *DEGS1* | AGCTAGTCTGCAAGCCACC | CTCTGGATACTTTGCCAGGAT |
| *CERS2* | GCTGGAGTCAGCCAAGATGT | AGGATCCAGAAGGGCAGGAT |
