# Supplementary material for: Inhibition of CERS1 in skeletal muscle exacerbates age-related muscle dysfunction
Source: eLife. 2024 Mar 20;12:RP90522. doi: 10.7554/eLife.90522 (PMC10954306; doi:10.7554/eLife.90522)
Supplement: Supplementary file 3. [file elife-90522-supp3.docx]

**Supplementary File 3.** List of plasmids.

| Plasmid | Reference |
| --- | --- |
| Lenti shRNA Cers1 | Origene # 93898 |
| psPAX2 | Addgene # 12260 |
| pMD2G | Addgene # 12259 |
