# Supplementary material for: Inhibition of CERS1 in skeletal muscle exacerbates age-related muscle dysfunction
Source: eLife. 2024 Mar 20;12:RP90522. doi: 10.7554/eLife.90522 (PMC10954306; doi:10.7554/eLife.90522)
Supplement: Supplementary file 4. [file elife-90522-supp4.docx]

**Supplementary File 4.** List of antibodies.

| Antibody | Supplier | Reference # |
| --- | --- | --- |
| Myosin 4 Monoclonal (MF20) | ThermoFisher Scientific | # 14-6503-82 |
| Anti-Myosin light chain 2 | Abcam | # ab79935 |
| Anti-Myosin light chain 1 | Thermofisher | # PA5-29635 |
| Laminin | Sigma | #L9393 |
| CD45 (1:200, eBioscience, eFluor450 conjugated) | eBioscience | # 48-0459-42 |
| Donkey anti-Mouse IgG secondary antibody | ThermoFisher Scientific | # A10037 |
| Donkey anti-Rabbit IgG secondary antibody | ThermoFisher Scientific | # A-21206 |
